# Poor Law Topics

**Published:** 1906-01-06

**Authors:** 


### POOR LAW TOPICS.

### THE LOCAL GOVERNMENT BOARD AND BOARDED-OUT CHILDREN.

THE dislike which some of the metropolitan unions have to boarding-out does not seem to be shared by the Local Government Board, who have recently issued a new order, which promises to make this method of dealing with children more generally useful than before. In the first place a new class of children may be so maintained. Hitherto the only children eligible were "orphans and deserted." To these may now be added those children who, owing to their parents' cruelty or neglect, are "adopted" by the Guardians. Of all the children who come under the Poor Law these are perhaps the most to be pitied, and if the foster-parents for them are wisely chosen their period of boarding-out should give them a new idea of the meaning of the word "home." One great objection to boarding-out in the unions of great cities was that, in order to find people who were willing to take in the children for a maximum payment of 4s. a week, it was necessary to go so far into the country that proper supervision could not be exercised over them. This has now been remedied in two ways. First, the maximum payment is raised to 5s. a week per child, at which rate it should not be difficult to find a home even in a place where rents are comparatively high. Secondly, the limit of distance from the residence of some member of the boarding-out committee is decreased from five miles to three. This will make visiting by the committee easier than it has hitherto been, and should ensure that frequent visitation which is so necessary to secure that the children are properly treated and to discover when they are not. Moreover, it is now provided that each boarded-out child and its home shall be visited at least once in six weeks. Another step in advance is the requirement that at least one member of the boarding-out committee shall be a woman, though as a matter of fact this is already very generally the case. Various restrictions are now introduced as to the persons to whom children may be entrusted. Thus it is forbidden to board out children in premises licensed for the sale of intoxicating liquors, or with any person who has been convicted of an offence which proves him to be unfit for the position of a foster-parent. Nor is a foster-parent allowed to insure a child's life for his or her own benefit. This new order should give an impetus to boarding-out in places where there has hitherto been some slackness in adopting it. We think, however, that still further extensions are necessary. Thus at present children cannot be boarded-out within their own union. In most Poor Law cases it is very necessary to break off all connection with the old environment, but cases occasionally do arise where an orphan child has a perfectly respectable relative within the union, who for a modest payment—probably less than the maximum allowance—would bring him up. Surely there should be some special discretion given to Guardians in such a case. Family bonds, where not in themselves undesirable, should surely be maintained. It is, however, difficult to make regulations which shall fit every possible contingency, and meanwhile we welcome the improvements shown in the new order.

